# Metformin Attenuates ROS *via* FOXO3 Activation in Immune Cells

**DOI:** 10.3389/fimmu.2021.581799

**Published:** 2021-04-19

**Authors:** Jelka Hartwig, Madlen Loebel, Sophie Steiner, Sandra Bauer, Zehra Karadeniz, Carsten Roeger, Carsten Skurk, Carmen Scheibenbogen, Franziska Sotzny

**Affiliations:** ^1^ Institute of Medical Immunology, Charité-Universitätsmedizin Berlin, Corporate Member of Freie Universität (FU) Berlin, Humboldt-Universität zu Berlin and Berlin Institute of Health (BIH), Berlin, Germany; ^2^ Science Center, Carl-Thiem-Klinikum Cottbus, Cottbus, Germany; ^3^ Department of Cardiology, Charité-Universitätsmedizin Berlin, Berlin, Germany; ^4^ DZHK (German Centre for Cardiovascular Research) Partner Site Berlin, Berlin, Germany; ^5^ Berlin-Brandenburg Center for Regenerative Therapies (BCRT), Berlin, Germany

**Keywords:** metformin, FOXO3, AMPK, reactive oxygen species, immune cells

## Abstract

Forkhead box O 3 (FOXO3) is a transcription factor involved in cell metabolism, inflammation and longevity. Here, we investigated if metformin can activate FOXO3 in human immune cells and affects the subsequent level of reactive oxygen/nitrogen species (ROS/RNS) in immune cells. AMP-activated protein kinase (AMPK) and FOXO3 activation were investigated by immunoblot or flow cytometry (FC) analysis, respectively. FOXO3 target gene expression was quantified by real-time PCR. ROS/RNS measurement using dichlorodihydrofluorescein diacetate (DCFH-DA) dye was investigated by FC. The role of the FOXO3 single nucleotide polymorphisms (SNPs) *rs12212067*, *rs2802292* and *rs12206094* on ROS/RNS production was studied using allelic discrimination PCR. Metformin induced activation of AMPK (pT172) and FOXO3 (pS413). ROS/RNS level was reduced in immune cells after metformin stimulation accompanied by induction of the FOXO3 targets mitochondrial superoxide dismutase and cytochrome c. Studies in Foxo3 deficient (*Foxo3^-/-^*) mouse splenocytes confirmed that metformin mediates its effects *via* Foxo3 as it attenuates ROS/RNS in myeloid cells of wildtype (WT) but not of *Foxo3^-/-^* mice. Our results suggest that FOXO3 can be activated by metformin leading to reduced ROS/RNS level in immune cells. This may add to the beneficial clinical effects of metformin observed in large cohort studies on longevity, cardiovascular and cancer risk.

## Introduction

Forkhead box O 3 (FOXO3) belongs to the family of FoxO transcription factors and is crucial in the regulation of cellular processes such as cell cycle regulation, apoptosis, cell metabolism, stress resistance and immunity (1). Single-nucleotide polymorphisms (SNPs) for *FOXO3* have been found to be associated with longevity (2, 3) and favorable outcomes in inflammatory disease (4). A role of FOXO3 in dampening immune reactions was discussed in several studies (5, 6). We showed that Foxo3 plays an important role in acute viral infection as its activation attenuated natural killer (NK) cell responses in a viral myocarditis model (7). Thus, FOXO3 modulation would have therapeutic potential in chronic and autoimmune diseases.

FOXO3 is phosphorylated by various kinases. Growth factor induced phosphorylation *via* phosphatidylinositol-4,5-bisphosphate 3-kinase (PI3K)/protein kinase B (Akt) pathway results in an increased binding to the regulator protein 14-3-3 and consequently in nuclear exclusion. This results in reduction of its transcriptional activity (1). The energy-sensing AMP-activated protein kinase (AMPK) mediated phosphorylation induces activation of FOXO3 (8). Moreover, acetylation/deacetylation and methylation are implicated in FOXO3 regulation (1).

Metformin (1,1-dimethylbiguanide hydrochloride) is the first-line oral drug for treatment of type 2 diabetes. It has been shown to reduce cardiac events in diabetic patients and improve the overall outcome and prognosis (9). *In vitro* metformin activates AMPK, which is upstream of FOXO3 (10). Further, a reduction of reactive oxygen/nitrogen species (ROS/RNS) in primary hepatocytes (11) and human monocytes and macrophages by metformin has been described (12, 13). Based on the favorable outcome of diabetic patients taking metformin, several studies are now investigating the influence of metformin on aging (targeting/taming aging with metformin, TAME study (14) and metformin in Longevity Study, MILES study, ClinicalTrials.gov Identifier: NCT02432287). We hypothesized that metformin is a drug that can activate FOXO3 in immune cells. Hence, the effect of metformin on FOXO3 activation, downstream targets and ROS/RNS level in immune cells was investigated in the present study.

## Materials and Methods

### Isolation of Human PBMCs

The study was approved and performed according to the ethical guidelines and regulations by the Institutional Ethics Committee (Charité Berlin). Informed consent was obtained from all subjects. Fresh PBMCs from healthy subjects were isolated from heparinized whole blood by density gradient centrifugation.

### Mice Studies

Splenocytes of FVB/N wildtype (WT) and FVB/N *Foxo3* deficient (*Foxo3^-/-^)* littermates were used. Experiments were conducted conform to the NIH Guide for the care and use of laboratory animals and were approved by regional authorities for provisions on labor, health, and technical safety, Berlin, Germany. WT and *Foxo3^-/-^* mice were sacrificed and spleens were taken. Single cell suspensions were made by passing the spleen through a cell strainer (BD) and diluting the suspension in cold PBS (4°C). The cells were cultured in RPMI 1640 containing l-glutamine and supplemented with 10% FCS, and 1% each of penicillin and streptomycin at 37°C, 5% CO_2_, or used immediately.

### AMPK Immunoblot

Freshly isolated human PBMCs were incubated for 3 h with metformin [0.01 - 10 mM]. The cells were then washed twice in ice cold PBS and solubilized in lysis buffer for 30 min on ice. After centrifugation for 10 min at 12000 x g and 4°C the supernatant was harvested. 20 µg of the protein lysate was separated on a gradient gel (7.5 -12.5%) and transferred to a nitrocellulose membrane. After blocking in 5% BSA for 1h, the membranes were incubated with the primary antibodies mouse-α-GAPDH (FF26A/F9) (1:10000, BioLegend), rabbit-α-AMPK (Thr172) 40H9 (1:500; Cell Signaling), mouse-α-AMPKalpha (F6) (1:500; Cell Signaling). The primary antibody was incubated overnight at 4°C in 5% BSA (in 0,05% TBS-T) and the secondary antibody goat-α-rabbit or α -mouse Ig-HRP 1:2000 (#7074/7076 Cell Signaling) was incubated for 1 h. Detection was achieved by using an enhanced chemiluminescence system (Pierce™ ECL Western Blotting Substrate) according to the manufacturer’s instructions. Quantification was performed by ImageJ software.

### Flow Cytometry for FOXO3 and ROS/RNS

CytoFLEX S, CytoFLEX LX (Backman Coulter), or LSR Fortessa (BD) was used. Data was analysed with FlowJo software 10.0.08. All gating strategies are shown in the **Supplementary Figure 1**. PBMCs were stained after stimulation with metformin [1 and 10 mM] or Compound C [CC, 10 µM] for 30 min at 37°C. 2% formalin fixed and 90% methanol permeabilized cells are blocked with 2% Flebo-γ. FOXO3 activation is visualized with an unlabeled monoclonal rabbit α -phospho-FOXO3 (Ser413) (D77C9) antibody (Cell Signaling) for 1 h. Secondary AF700-labelled goat-α-rabbit antibody (Invitrogen) was incubated for 30 min. Cell surface molecules CD3-PB (UCHT1; BioLegend), CD56-PE (HCD56; BioLegend), and CD14-FITC (HCD14; Biolegend) are stained afterwards for 15 min at 4°C.

Intracellular ROS/RNS staining was performed following 3 h metformin stimulation [0.1 - 20 mM]. For this, cells were stained with CD3-Per.CP-Cy5.5 (SK7); CD14-APC (M5E2); CD19-PE-Cy7 (HIB19) and CD56-PE (HCD56) purchased from BioLegend. After 10 min stimulation with phorbol 12‐myristate 13‐acetate (PMA) (Sigma Aldrich) [100 ng/ml] as positive control, cells were stained with viable and dead cells LIVE/DEAD™ Fixable Aqua dead cell stain kit (Life Technologies) followed by dichlorodihydrofluorescein diacetate (DCFH-DA) [5 µM; Sigma-Aldrich] incubation for 30 min. Mouse ROS/RNS measurement was performed according to the same protocol using staining antibodies (CD3-AF700 (17A2; Invitrogen); CD19-APC (6D5; BioLegend); Nkp46-APC (29A1.4; BioLegend); CD11b-PE (M1/70; eBioscience); CD45-PE-Cy7 (30-F11; eBioscience); Ly6C-PerCPCy5.5 (HK1.4; Invitrogen).

### PCR

Total RNA was extracted (RNeasy^®^ Mini Kit QIAGEN^®^) according to the manufacturer’s instructions. cDNA was prepared by reverse transcription and real-time PCR was performed using TaqMan^®^ Universal PCR Master Mix (Applied Biosystems) and TaqMan^®^ Gene Expression Assays for *SOD2, HMOX1, CYCS* and 18S rRNA (Applied Biosystems). All analyses were performed with the ABI7200 and software Step One Plus as absolute quantification according to manufacturer’s instruction. Relative expression was analysed using the ΔΔCT method and normalized against 18S rRNA.

### SNP Analysis

Genomic DNA was isolated (QIAmp^®^ DNA Blood Mini Kit QIAGEN^®^) according to the manufacturer’s protocol. Analysis of the FOXO3 SNP *rs12212067* (ID: C:30780203_10), SNP *rs2802292* (ID: C:16097219_10) and SNP *rs12206094* (ID: C:30780173_10) was performed by allelic discrimination PCR according to the manufacturer’s instruction using Applied Biosystems 7500 Fast Real-Time PCR system. 10 ng of genomic DNA were used in a PCR reaction. TaqMan^®^ SNP Genotyping Assays were purchased from Applied Biosystems. SNP genotypes are shown in the **Supplementary Table**.

### Statistical Analysis

Statistical data analysis was carried out using GraphPad Prism software, version 6.0. and 7.0 Continuous variables were expressed as median and interquartile range, if not indicated otherwise. Univariate comparison of two independent groups were done using Mann-Whitney U test (Mann Whitney test) and of dependent groups with the Wilcoxon matched-pairs signed rank test (Wilcoxon test). P values were calculated in a two-tailed manner. In all cases, the significance level was set to *P value < 0.05, **P < 0.01.

## Results

### Metformin Activates FOXO3 in an AMPK Dependent Manner in Human Immune Cells

**Figure 1:**
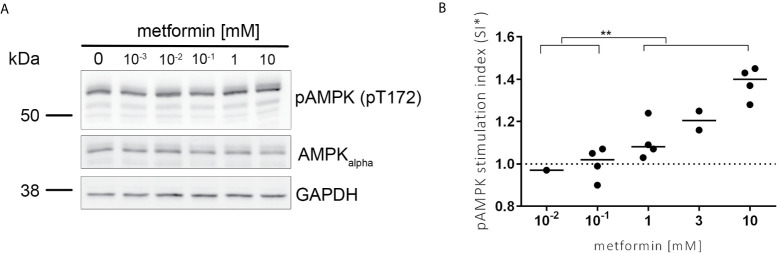
AMPK is activated in a dose dependent manner by metformin. **(A)** Representative image of the protein amount of pAMPK (pT172), AMPKalpha and GAPDH analysed in lysates of PBMCs stimulated for 3 h with metformin using specific antibodies by immunoblot analysis. pAMPK (pT172) and AMPKalpha are run on two separate gels in parallel each with GAPDH as control. **(B)** Activating phosphorylation of AMPK was quantified using Image J (NIH). The median stimulation index (SI, treated/untreated) is shown for the pAMPK/AMPKalpha ratio normalized to unstimulated control. Significance was calculated with the Mann-Whitney test between indicated groups. **P ≤ 0.01.

To investigate the activation of FOXO3, we first studied the effect of metformin on the AMPK kinase upstream of FOXO3 by immunoblot. We observed a dose dependent increase of the activating phosphorylation of AMPK (pT172) after incubation with metformin in human blood immune cells (**Figure 1**).

**Figure 2:**
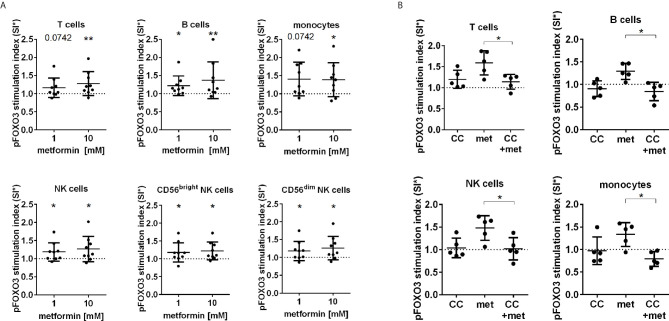
FOXO3 is activated by metformin in an AMPK dependent manner. **(A)** FOXO3 (pS413) expression in T cells, B cells, monocytes and NK cells (total, CD56dim and CD56bright) after 30 min stimulation analysed by flow cytometry. **(B)** Addition of the AMPK inhibitor Compound C (CC) [10 µM] abrogated the effect of 10 mM metformin. SI: The MFI (AF700) of FOXO3 (pS413) expression subtracted by background signal (GAR) is depicted in relation to the unstimulated control. Median with interquartile range is shown. Wilcoxon test is used for statistical analysis in relation to unstimulated control or between indicated groups. *P ≤ 0.05, **P ≤ 0.01.

Next, the effect of metformin on the activating phosphorylation of FOXO3 (pS413) was studied in blood immune cells by FC. We observed an activation of FOXO3 by upregulation of pS413 in all human immune cell subpopulations including T cells, B cells, monocytes and both CD56^bright^ and CD56^dim^ NK cells after metformin incubation (**Figure 2A**). To elucidate the involvement of AMPK in metformin induced FOXO3 activation, AMPK was additionally inhibited by Compound C. The inhibition of AMPK resulted in a significant reduction of metformin induced phosphorylation of FOXO3 in all immune cells (**Figure 2B**).

### Metformin Attenuates ROS/RNS Production

**Figure 3:**
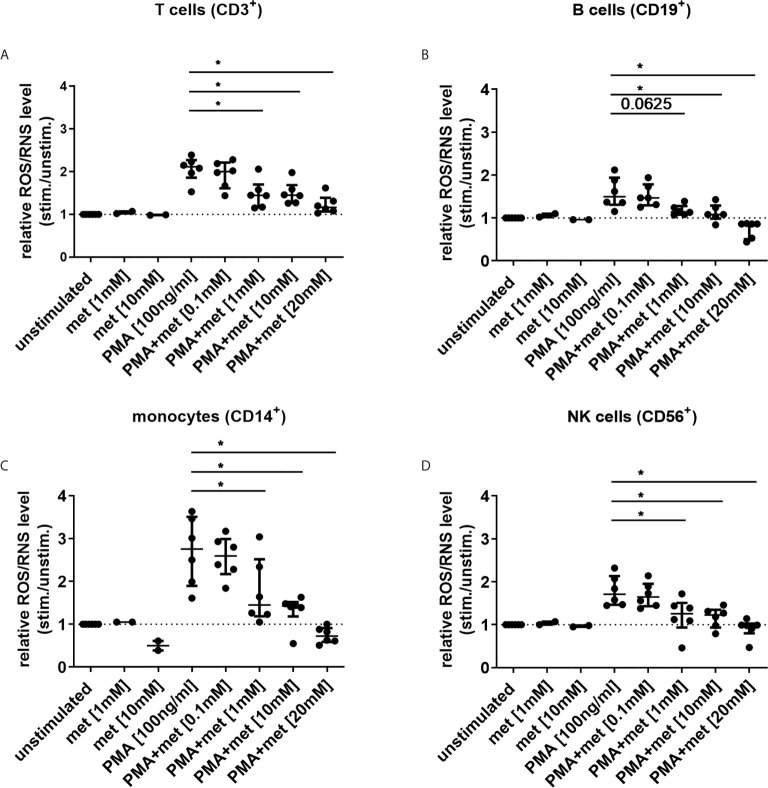
Metformin reduces ROS/RNS level in human immune cells in a dose-dependent manner. **(A–D)** ROS/RNS level was measured in blood immune cells, cultured with metformin for 3 h (n=6; met alone n=2). Cells were either unstimulated or stimulated with PMA [100 ng/ml] for 10 min and ROS/RNS generation was measured by FC in **(A)** T cells, **(B)** B cells, **(C)** monocytes and **(D)** NK cells using DCFH-DA. Median with interquartile range is depicted and Wilcoxon test is used for statistical analysis. *P ≤ 0.05.

Next, the effect of metformin on ROS/RNS level was studied in immune cells under similar conditions. PMA-induced ROS/RNS level was diminished by metformin in CD14^+^ monocytes, CD3^+^ T cells, CD19^+^ B cells and CD56^+^ NK cells in a dose-dependent manner (**Figures 3A–D**). In unstimulated CD14^+^ monocytes ROS/RNS level was already diminished when incubated with 10 mM metformin (**Figure 3C**).

**Figure 4:**
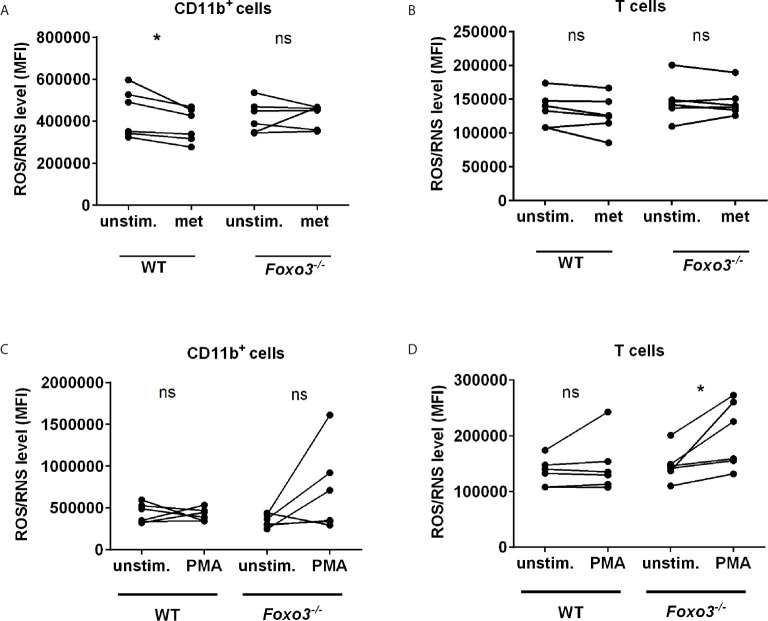
ROS/RNS production is reduced by metformin in WT and induced by PMA in *Foxo3^-/-^* mice. ROS/RNS level was analysed in WT vs. *Foxo3^-/-^* splenocytes after 3 h of metformin [0.1 mM] **(A, B)** or 10 min of PMA [100 ng/ml] treatment **(C, D)**. Mouse splenocytes were labeled with DCFH-DA and ROS/RNS level was analysed by FC (n=6) in **(A, C)** CD11b^+^ myeloid cells and **(B, D)** CD3^+^ T cells. Significance was evaluated using the Wilcoxon test. *P ≤ 0.05; ns, not significant.

To get further evidence that metformin attenuates ROS *via* FOXO3 we comparatively analysed WT and *Foxo3^-/-^* mice. As observed in human monocytes, incubation of splenocytes with metformin diminished ROS/RNS level in WT CD11b^+^ monocytes. In contrast metformin had no effect on monocytes in ROS/RNS in *Foxo3^-/-^* mice, providing further evidence that Foxo3 is involved in this pathway (**Figure 4A**). ROS/RNS level in CD3^+^ T cells of WT mice were not reduced by metformin (**Figure 4B**). Stimulation of splenocytes with PMA leads to an increased ROS/RNS production in 3 of 5 *Foxo3^-/-^* mice in CD11b^+^ (not significant) and in all 5 *Foxo3^-/-^* mice in T cells (p=0.03) (**Figures 4C, D**). Interestingly, the rather short incubation time of 10 min with PMA chosen to avoid toxicity in *Foxo3^-/-^* mice was not sufficient to induce the ROS/RNS production in WT mice.

### Metformin Promotes Gene Expression of Antioxidative Enzymes and the Autophagic Marker LC3

**Figure 5:**
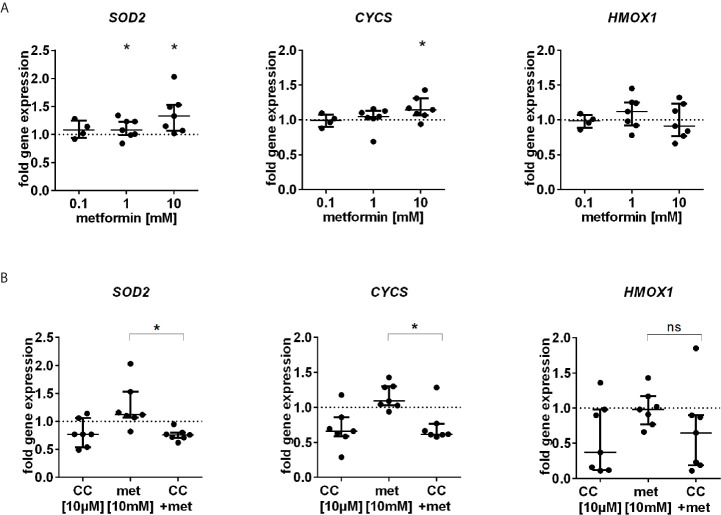
Gene expression of antioxidative enzymes is induced by metformin. **(A)** RNA expression analysis of *SOD2, CYCS and HMOX1*, three FOXO3 target genes after 3 h incubation with different dosages of metformin (n=4-7). **(B)** Antioxidative gene expression by metformin is AMPK dependent. Gene expression analysis of *SOD2, CYCS* and *HMOX1* after 3 h incubation with metformin [10 mM] alone or in combination with CC [10 µM] (n=7). Gene expression was normalized to 18S rRNA and is depicted as fold induction to unstimulated control. Median with interquartile range is shown and significance was evaluated between unnormalized data and refers to unstimulated control using the Wilcoxon test. *P ≤ 0.05; ns, not significant.

We further studied the expression of FOXO3 regulated antioxidative enzymes SOD2, CYCS and HMOX1. Metformin stimulated the gene expression of *SOD2* and *CYCS* but not of *HMOX1* (**Figure 5A**). The enhanced gene expression was abolished by coincubation with the AMPK inhibitor Compound C (**Figure 5B**).

In addition, we studied the gene expression of *MAP1LC3A* coding for the autophagic marker LC3. The gene expression was induced in PBMCs treated with 10 mM metformin compared to untreated control (p=0.047) (**Figure S2**). However, this increase was not observed in cells co-treated with Compound C, implicating AMPK in FOXO3 dependent autophagic gene regulation by metformin.

### *FOXO3* Gain-of-Function SNPs Do Not Influence ROS/RNS Level in Human Immune Cells

**Figure 6:**
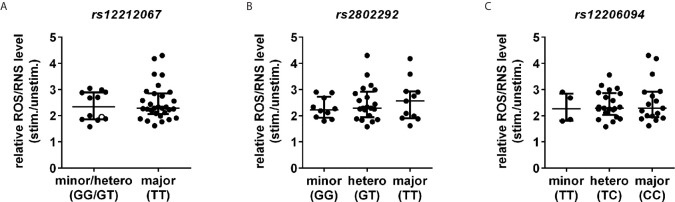
*FOXO3* gain-of-function SNPs have no influence on ROS/RNS level. ROS/RNS level in CD14^+^ monocytes were investigated following stimulation with PMA [100 ng/ml] for 10 min by DCFH-DA staining using FC. **(A)** *rs12212067* SNP carrier minor/heterozygous n=12 and major n=30. Minor allele carrier is depicted with an open circle (○). **(B)** *rs2802292* SNP carrier minor n=10, heterozygous n=21 and major n=11 and **(C)** *rs12206094* SNP carrier: minor n=4, heterozygous n=21 and major n=17. Median with interquartile range is depicted and Mann-Whitney test is used for statistical analysis between independent cohorts.

Finally, the three different gain-of-function *FOXO3* SNPs *rs12212067*, *rs2802292* and *rs12206094* associated with aging (2, 3) and inflammation (4) were analysed for their potential influence on ROS/RNS level in immune cells of healthy controls. We observed neither differences in the ROS/RNS level in monocytes at baseline (data not shown) nor after 10 min of PMA stimulation in carriers of the longevity/immune dampening-associated alleles of the SNPs (**Figure 6**).

## Discussion

*FOXO3* is a gene associated with healthy aging in centenarians, a lower prevalence of cardiovascular events and a better outcome in inflammatory disorders (2–4, 15). Therefore, it is of great interest to find drugs that activate FOXO3 and target downstream ROS and inflammatory cytokine responses. Here, we provide evidence that metformin activates FOXO3 *via* the AMPK signalling pathway which subsequently leads to the induction of FOXO3 antioxidative target genes *SOD2* and *CYCS* as well as the induction of autophagic gene expression. Further we show that triggering this pathway by metformin is associated with reduced ROS/RNS level. To our knowledge this is the first study providing evidence that FOXO3 can be activated by metformin in immune cells.

We found that FOXO3 is activated by metformin stimulation in immune cells. Using the AMPK inhibitor Compound C we provide evidence that metformin-triggered FOXO3 activation is AMPK-dependent. However, Compound C has been shown to inhibit several other kinases, including the FOXO3 regulator Akt (16). We cannot thus exclude the possibility that other kinases might have been affected by Compound C in our experiments. However, in line with our findings a metformin-triggered, AMPK-dependent activation of FOXO3 was shown previously by Chou et al. in cancer cells by targeting the Akt-MDM2-FOXO3 signalling axis (17). Further, we provide evidence in our study that FOXO3 is phosphorylated at an AMPK specific phosphorylation site, Serine 413, following metformin incubation. Furthermore, metformin activates gene expression of FOXO3 targets *SOD2* and *CYCS*. The upregulation was diminished by co-treatment with the AMPK inhibitor Compound C indicating an involvement of AMPK function. In addition, metformin incubation resulted in reduced PMA-stimulated ROS/RNS production in human immune cells. Mitochondrial *SOD2* is important for neutralizing ROS. The enzyme binds to superoxide byproducts of oxidative phosphorylation and converts them to hydrogen peroxide and diatomic oxygen (18). CYCS is a small hemeprotein found loosely associated with the inner membrane of the mitochondrion. It belongs to the cytochrome c family of proteins and can remove superoxide (O_2_^–^) and H_2_O_2_ from mitochondria (19). Reduction of ROS/RNS levels in human cells by metformin has already been shown by other groups for other cell types including primary hepatocytes and vestibular cells (11, 20). The study of Hou et al. indicates that metformin reduces ROS by inducing Trx expression *via* AMPK-FOXO3 signalling in aortic endothelial cells (21). In addition, we observed an increased gene expression of LC3, an autophagic marker, in metformin treated, but not in Compound C co-treated cells. These data suggest, that metformin might induce autophagy in PBMCs probably in an AMPK-dependent manner. Autophagy is an essential mechanism in response to oxidative stress and associated mitochondrial dysfunction (22). It enables the clearance of damaged proteins and cell organelles. Interestingly, the LC3 gene is described as target gene for FOXO3 (23) and it was shown in several studies that autophagy can be induced *via* AMPK-FOXO3 signalling (24–26). These data corroborate our results about the effects of metformin-AMPK-FOXO-signalling in immune cells. In a recent study by Gillespie et al. it was shown that metformin stimulation results in decreased fibroblast proliferation and increased *FOXO3* promotor occupancy. However, pathway analysis of FOXO3 activation and downstream effects were not studied (27).

Using *Foxo3^-/-^* mice we provide further evidence that the reduction of ROS/RNS levels by metformin at least partly depends on Foxo3 as we observed that metformin reduced basal intracellular ROS/RNS level in WT but not *Foxo3^-/-^* mice. A metformin dependent reduction of the mitochondrial ROS production by direct inhibition of mitochondrial complex I was discussed in several studies (28). We cannot exclude a partial direct effect of metformin on the mitochondrial ROS production in our experiments. As metformin-induced antioxidative gene expression was observed in our study we provide evidence about an AMPK-FOXO3-axis dependent cellular stress response also involved in the decline in ROS level. In addition, our experiments using *Foxo3^-/-^* mice further confirm the antioxidative properties of Foxo3, as PMA enhanced ROS/RNS in CD11b^+^ and T cells of *Foxo3^-/-^* mice but not WT mice. This is in line with the study of Joseph et al. Here the ROS production, induced by a chronic *Salmonella typhimurium* infection, was more prominent in splenocytes of *Foxo3a^−/−^* when compared to WT mice (29). Furthermore, they observed a decreased expression of antioxidative genes in infected *Foxo3a^−/−^* mice. In general, the important role of FOXO3 in the intracellular oxidative stress response and damage repair *via* gene expression of antioxidative as well as autophagy-related genes has been determined in several studies (1, 23).

SNPs in *FOXO3* were shown in previous studies to be associated with ameliorated clinical course of rheumatoid arthritis (30), Crohn´s disease (4) and viral myocarditis (7). In the study by Lee et al. monocytes from healthy subjects carrying the SNP *rs12212067* had lower TNF-α response upon LPS stimulation (4). In our previous study, healthy subjects with the *FOXO3* SNP *rs12212067* had lower IFN-γ production in peripheral NK cells after 18 h of R848 stimulation (7). Furthermore two of the here analysed SNPs (*rs2802292*, *rs12206094*) are associated with longevity (2, 3). In the present study we therefore wanted to investigate if these three SNPs, located within different intronic regions with a low linkage disequilibrium, have an influence on ROS/RNS level in human immune cells and if so may influence the response to metformin. Grossi et al. showed that the SNP *rs2802292* is associated with diminished ROS content, measured with DCFH-DA in human primary dermal fibroblasts after stressing the cells with H_2_O_2_. They found that the SNP, located in the intron 2 of *FOXO3* influences enhancer function creating a novel binding site for HSF1, so inducing *FOXO3* expression along with stress response *via* upregulation of target genes (*SOD2, CAT, GADD45A, HSPA1A*) (31). In contrast we could not observe an association of any of the 3 SNPs with levels of ROS/RNS in immune cells.

We have evidence from our previous study that FOXO3 plays a role in attenuating cytokine responses. We could show that NK cells of *Foxo3^-/-^* mice had an increased IFN-γ response to Il-2. Furthermore, healthy carrier of the immune dampening-associated *FOXO3* SNP *rs12212067* had reduced IFN-γ producing CD56^bright^ NK cells after 18 h of R848 stimulation (7). Diminished levels of ROS itself may play a role in attenuation of cytokine levels as it was shown that ROS activates NF-κB and TH1 cytokine production (32). Thus, FOXO3 may affect cytokine production by reducing intracellular ROS. However, other studies showed that metformin can inhibit NF-κB directly (33). Further, it was shown that FOXO3 antagonizes signalling intermediates downstream of the Toll‐like receptor (TLR) 4, such as NF‐κB and IRFs, resulting in decreased IFN‐β expression in human monocyte‐derived DCs (34). Buldak et al. showed that metformin in a dose of 0.02 and 2 mM reduces the LPS-induced TNF-α response in human monocyte derived macrophages after 24 h (13). We could not observe an influence of metformin on LPS induced TNF-α production in human monocytes under similar conditions (data not shown).

In conclusion, our data provide evidence that metformin activates FOXO3 in immune cells resulting in reductions of ROS/RNS stress. This effect may be beneficial in chronic inflammation and atherosclerosis and may play a role in the improved morbidity and mortality of diabetes patients taking metformin (9). Ongoing trials (TAME, MILES) are examining the effect of metformin on morbidity and mortality in healthy older people (ClinicalTrials.gov Identifier: NCT02432287 (14). Based on our findings it would be very interesting to study the effect of metformin on FOXO3 activation in clinical trials and the potential influence of *FOXO3* SNPs.

## Data Availability Statement

The raw data supporting the conclusions of this article will be made available by the authors, without undue reservation.

## Ethics Statement

The studies involving human participants were reviewed and approved by Institutional Ethics Committee (Charité Berlin). The patients/participants provided their written informed consent to participate in this study. The animal study was reviewed and approved by Landesamt für Gesundheit und Soziales (LAGeSo).

## Author Contributions

Funding Acquisition: ML, CSc and CSk. Conceptualization: JH, FS, ML and CSc. Investigation: JH carried out the experiments with the exception that SS performed DCFH-DA staining in healthy controls. Writing-Original Draft Preparation: JH, CSc and FS. Resources: SB contributed to materials and reagents, ZK and CR helped designing the mice study and provided samples. Formal Analysis: JH, FS and CSc. Writing-Review and Editing: FS, ML, ZK, CR, CSc and CSk. All authors contributed to the article and approved the submitted version.

## Funding

This study was supported by a Grant of the German Research Council (DFG) (SCHE 478/9-1) to CSc and CSk.

## Conflict of Interest

The authors declare that the research was conducted in the absence of any commercial or financial relationships that could be construed as a potential conflict of interest.
