# Supplementary material for: Metformin Attenuates ROS via FOXO3 Activation in Immune Cells
Source: Front Immunol. 2021 Apr 19;12:581799. doi: 10.3389/fimmu.2021.581799 (PMC8089390; doi:10.3389/fimmu.2021.581799)
Supplement: Supplementary file 1 [file DataSheet_1.pdf]

Supplementary Material

1 Supplementary Method

Analysis of *MAP1LC3A* gene expression

Total RNA was extracted (RNeasy® Mini Kit QIAGEN®) according to the manufacturer's instructions. cDNA was prepared by reverse transcription and real-time PCR was performed using TaqMan® Universal PCR Master Mix (Applied Biosystems) and TaqMan® Gene Expression Assays for *MAP1LC3A* and 18S rRNA (Applied Biosystems). Analyses was performed with the ABI7200 and software Step One Plus as absolute quantification according to manufacturer's instruction. Relative expression was analyzed using the ΔΔCT method and normalized against 18S rRNA.

Univariate analysis was performed using Wilcoxon matched-pairs signed rank test (Wilcoxon test). P values were calculated in a one-tailed manner.

2 Supplementary Figures

2.1 Supplementary Figure 1

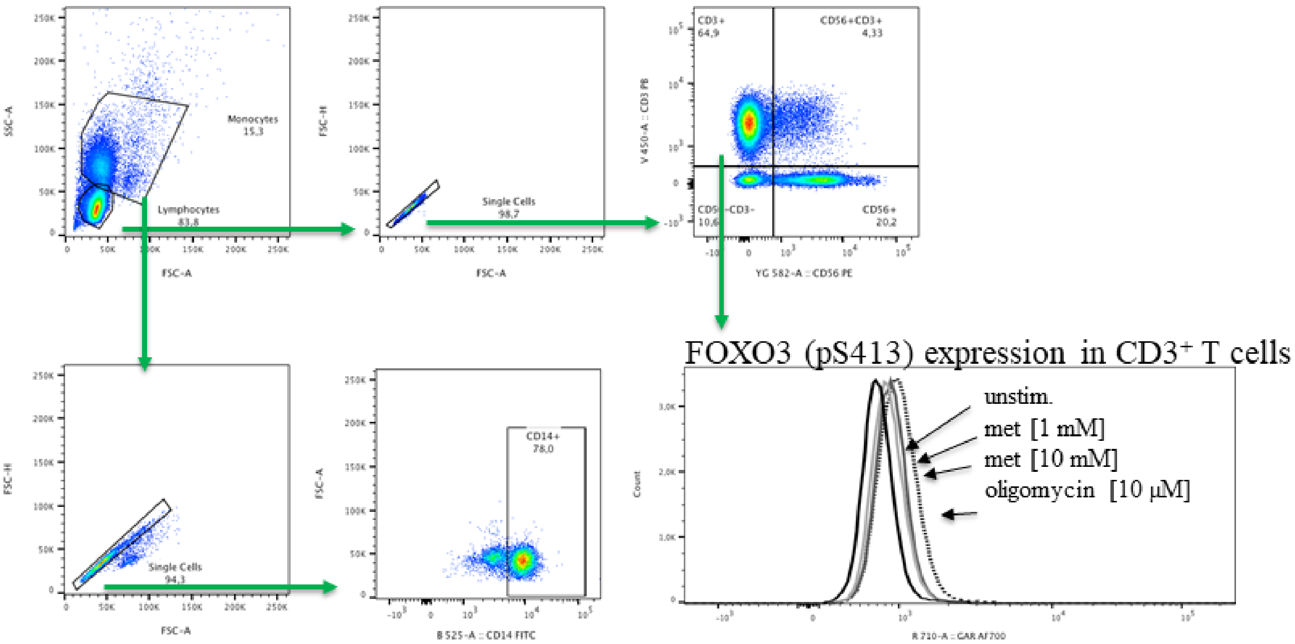

Figure S1: Representative gating strategy along with FOXO3 (pS413) expression in $CD3^+$ T cells. Histogram is showing FOXO3 (pS413) expression after 30 min of stimulation with metformin [1mM, 10 mM] and oligomycin [10 µM].

2.2 Supplementary Figure 2

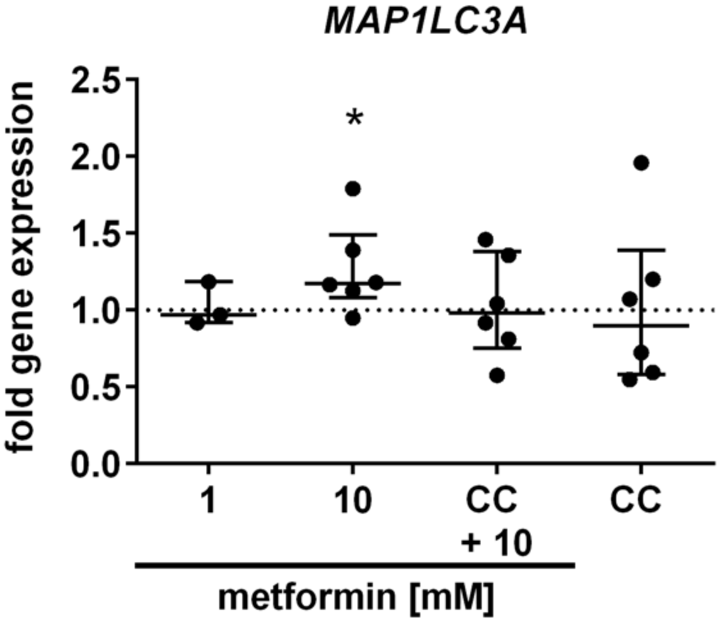

Figure S2: Gene expression of autophagic marker LC3 is induced by metformin. (A) RNA expression analysis of the gene *MAP1LC3A* after 3 h incubation with different dosages of metformin alone or in combination with Compound C (CC) [10 µM] (n=3-6). Gene expression was normalized to 18S rRNA and is depicted as fold induction to unstimulated control. Median with interquartile range is shown and significance was evaluated using a one-tailed Wilcoxon test. *P ≤ 0.05

3 Supplementary Table

Nr.	rs12212067	rs2802992	rs12206094
1	T/T	G/G	C/C
2	T/T	G/T	C/T
3	T/T	G/T	C/T
4	T/T	T/T	C/C
5	T/T	T/T	C/C
6	T/T	G/G	C/T
7	T/T	G/T	C/C
8	G/T	G/T	C/T
9	G/T	G/G	T/T
10	T/T	G/T	C/T
11	G/T	G/T	C/T
12	T/T	G/T	C/T
13	T/T	G/T	C/T
14	G/G	G/G	C/T
15	T/T	G/T	C/T
16	T/T	T/T	C/C
17	T/T	G/T	C/T
18	G/T	G/G	T/T
19	T/T	T/T	C/C
20	G/T	G/T	C/C
21	T/T	G/T	C/T
22	G/T	G/T	C/T
23	T/T	T/T	C/C
24	T/T	T/T	C/C
25	G/T	G/G	C/T
26	G/T	G/T	C/C
27	T/T	G/T	C/T
28	T/T	G/T	C/C
29	T/T	G/T	C/T
30	T/T	G/T	C/C
31	T/T	G/G	C/T
32	G/T	G/T	C/T
33	T/T	T/T	C/C
34	T/T	T/T	C/C
35	T/T	T/T	C/C
36	G/T	G/G	T/T
37	T/T	G/G	C/T
38	T/T	G/T	C/T
39	G/T	G/T	C/T
40	T/T	G/G	T/T
41	T/T	T/T	C/C
42	T/T	T/T	C/C
	T/T n=30<br>G/T n=11<br>G/G n=1	T/T n=11<br>G/T n=21<br>G/G n=10	C/C n=17<br>C/T n=21<br>T/T n=4
